# Isopathy

**Published:** 1848-06

**Authors:** 


*Isopathy.*—A new medical doctrine has appeared on the horizon, and it is Germany again, *alma parens rerum,* which enriches the world with this benefit. Homœopathy, magnetism, and phrenology salute their new sister under the harmonious name of Isopathy. Dr. Hermann is the prophet of this doctrine, which is based on the following principle :—Every diseased organ has its remedy in the same organ—thus, if you have a disease of the liver, eat liver ; if a headache, eat brain ; if you suffer in the bladder or kidneys, nourish yourself on bladder and kidneys ; if the testicle be disordered, eat testicle. As the organs may not appear very tempting to certain squeamish persons, M. Hermann has made tinctures of them, which his patients take in spoonfuls, under the scientific names of stomachine, cystine, testiculine, unbria, &c. The work published at Augsburgh contains fifty cases of radical cures. Go, young doctrine, increase and prosper—thou wilt doubtless be called to high destinies !—*Medical Gazette.*

